# Intrapersonal and interpersonal level factors influencing self-care practices among Hong Kong individuals with COVID-19—A qualitative study

**DOI:** 10.3389/fpubh.2022.964944

**Published:** 2022-08-24

**Authors:** Haixia Ma, Yajing Ma, Song Ge, Shanshan Wang, Ivy Yan Zhao, Martin Christensen

**Affiliations:** ^1^School of Nursing, The Hong Kong Polytechnic University, Hung Hom, Kowloon, Hong Kong SAR, China; ^2^Interdisciplinary Centre for Qualitative Research, School of Nursing, The Hong Kong Polytechnic University, Hung Hom, Kowloon, Hong Kong SAR, China; ^3^School of Public Policy and Management, China University of Mining and Technology, Xuzhou, Jiangsu, China; ^4^Department of Natural Sciences, University of Houston-Downtown, Houston, TX, United States; ^5^WHO Collaborating Center for Community Health Services, School of Nursing, The Hong Kong Polytechnic University, Hung Hom, Kowloon, Hong Kong SAR, China

**Keywords:** self-care, COVID-19, home quarantine, intrapersonal, interpersonal

## Abstract

**Background:**

The unprecedented crisis during the fifth wave of the COVID-19 pandemic in Hong Kong placed a significant burden on the health care system. Therefore, the Hong Kong government advocated that individuals with no or mild COVID-19 symptoms should self-care at home. This study aimed to understand intrapersonal and interpersonal level factors that shaped self-care practices among home-quarantined individuals with COVID-19 during the peak of the pandemic.

**Methods:**

This study used convenience and snowball sampling whereby a total of 30 semi-structured telephone interviews were conducted between March and April 2022. Inductive content analysis was used to analyze the data.

**Results:**

Factors reported at the intrapersonal level included socioeconomic status and housing conditions, information and knowledge about COVID-19, long COVID, and psychological adjustments brought about by home quarantine. Factors identified at the interpersonal level included caregiving responsibilities, family relationships, and social support.

**Conclusions:**

Findings from this study identified a combination of intra and interpersonal level factors influenced an individual's self-care practices as a result of pandemic-induced quarantine. It was particularly concerning for those individuals in socially and economically deprived groups, where access to services was challenging. This study also raised awareness of the ineffectual and insufficient knowledge individuals held of self-medication and overall COVID-19 management. A key recommendation is developing family-based resilience programmes to support and empower vulnerable families to better cope with the realities of self-quarantine.

## Introduction

The term “self-care,” is defined by the World Health Organization (WHO) as “the ability of individuals, families, and communities to promote health, prevent disease, maintain health, and to cope with illness and disability with or without the support of a health-care provider” (1). It usually encompasses a number of factors, for example, health information, medication usage, symptom management, management of mental health issues, lifestyle and social support, and communication (2). Self-care is considered a cost-effective solution as it plays a crucial role in reducing health care utilization and its associated costs and empowers individuals to take a more proactive role in supporting their own health needs. Self-care has been increasingly implemented in chronic disease management and more recently pandemic control (2–4).

Self-care management has been implemented as a key decisive strategy to help combat the COVID-19 pandemic in many countries since 2020. According to international guidelines, self-care management for mild or moderate COVID-19 is focused on symptom management and the prevention and/or reduction in virus transmission (5–8). Specifically, the recommendations also included:

- The implementation of Rapid Antigen Testing (RAT) to lessen the burden (including costs) on Polymerase Chain Reaction (PCR) testing undertaken by health care facilities,
- Making individuals more aware in recognizing the impending emergency warning signs (for example, increasing shortness of breath),
- An emphasis on maintaining “environmental hygiene” (for example health housing),
- Increased vigilance in promoting protective measures for family members,
- The management of daily necessities (such as food and access to medications), and
- A detailed plan to supporting quarantine measures including time in quarantine.

## The Hong Kong context

As a Special Administrative Region of the People's Republic of China, Hong Kong has a population of approximately 7.4 million. Hong Kong started to adopt a self-care management approach during its fifth and worst wave since the initial COVID-19 outbreak. Although the city has successfully managed four waves with multiple policies, like those employed by other countries such as, social distancing, mandatory mask-wearing, and tight border controls (9), the fifth wave of COVID-19 infection (dominated by the Omicron variant with the first cases reported in December 2021) significantly affected the Hong Kong population. During the first week of March for example, the city recorded approximately 80,000 cases/day and 150 COVID-related deaths, at the time this was the highest fatality rate among developed countries (10). By mid-May 2022, the accumulated number of positive cases since the outbreak of the fifth wave had surpassed 1.2 million people (11).

The unprecedented crisis during the fifth wave of the COVID-19 pandemic placed a significant burden on the health care system and those families of individuals with COVID-19. Overwhelmed by the sharply increasing number of individuals testing positive for the virus, the public hospital system was constantly challenged to provide effective and efficient care and was in imminent danger of collapse. For reasons unknown, some private hospitals, refused to provide care for individuals with COVID-19 (12) and therefore, to alleviate the pressure on the public hospital system, the Hong Kong government adjusted its guidelines on testing, quarantine, and treatment for those testing positive. In line with the WHO emergency guidelines and strategies applied in many affected countries (5, 13), the Hong Kong government launched the “StayHomeSafe” scheme and advocated that individuals testing positive and with no or mild symptoms should practice self-care at home (14). To avoid duplication of resources and the inherent time delays, individuals testing positive using the RAT could register through an online system without seeking further confirmation of their test results from hospitals (15). To complement this information, hotlines had been established for those seeking additional information regarding symptom enquiries, especially for those patients who were waiting to be admitted to hospital for treatment or to quarantine facilities. In addition, a number of designated community-based clinics (Community Testing Centers) had been set up to provide free of charge PCR testing for those people with mild symptoms and those who were required by law to get tested (16).

## Pandemic induced self-care

Although self-care management has been considered an important strategy during the outbreak of the COVID-19 pandemic, until now, international studies evaluating the effectiveness and implementation of self-care management programmes for COVID-19 are few in number (17–20), and most of them appear to focus on an individuals' psychological well-being. The self-care experience among Hong Kong individuals with COVID-19 during home quarantine remains unknown. A better understanding of an individuals' self-care practices and concerns during home quarantine, and the influences these may have on their family members could inform the development of policy and services to promote specific self-care programmes and prepare for possible future epidemic/pandemic outbreaks. Therefore, this qualitative study aimed to understand the individual and interpersonal level factors that shaped self-care practices among home-quarantined individuals with COVID-19 amid the fifth wave of the COVID-19 in Hong Kong.

## Materials and methods

### Study design

This qualitative study was part of a larger study exploring the experiences of home-quarantined individuals during the fifth wave of the COVID-19 pandemic in Hong Kong. Individual telephone interviews were conducted among 30 home-quarantined individuals who tested with COVID-19 in Hong Kong.

### Participants and recruitment

Home-quarantined individuals testing positive with COVID-19 in Hong Kong were recruited using convenience and snowball sampling methods to ensure a variety of age groups were included in the sample. Data saturation was reached from 30 participants. Among the participants, 17 were recruited through the research team's social networks, and the remaining 13 were recruited through snowball sampling from those already interviewed.

To be eligible for the study, participants: (1) were over 18 years of age; (2) had been tested positive with COVID-19 either through RAT or nucleic acid test from February 2022, which also included individuals with any clinical symptoms or who were in recovery; (3) were quarantined at home; (4) were able to speak Cantonese/Putonghua/English; (5) were willing to participate in the study and share their experience via telephone or video calls (e.g., Zoom, WhatsApp, FaceTime); and (6) were able to give informed consent.

Participants were excluded if they: (1) had been diagnosed with a significant mental health issue or mood disorders, such as anxiety disorders, depression, psychosis, or bipolar disorders; and (2) had a hearing, communication, or cognitive impairment.

### Data collection

Due to the social distancing measures and the nature of the pandemic, 30 semi-structured telephone interviews were held between March to April 2022. The telephone interviews were conducted at a mutually convenient time for the participants and the researcher. Each participant was sent a participant information sheet prior to the telephone interview via email or text message. Before each interview, the researcher explained the aims and purpose of the study and why they were being asked to participate. The participant's anonymity, confidentiality, and voluntary participation of the interview were assured. Once the participants agreed to participate and provided verbal consent, a telephone interview was scheduled based on their availability. At the time of the interview, the content of the information sheet was re-addressed and verbal consent for audio-recording the interview was obtained and recorded prior to the commencement of the interview. Open-ended questions were used to identify those factors deemed important by individuals practicing self-care as a result of home quarantine with COVID-19. Questions, such as “How did you manage the symptoms?” “How did you practice home quarantine” were used, and factors at an intra and interpersonal level that influenced their experiences were explored. Prompts, such as “Tell me more about what it was like” and “Why do you think that” were used. The details of the interview guide are shown in the Supplementary material (Appendix I). The interviews lasted on average of 34 min. After the interview, each participant received a HKD$50 (≈USD 6.37) supermarket coupon in appreciation of their participation.

All interviews were conducted by a female bilingual researcher (HXM) in Mandarin Chinese or Cantonese. The research team consisted of a postdoctoral fellow and associate professor both of whom have extensive experience in conducting qualitative research studies.

### Data analysis

The interview data was analyzed using inductive content analysis as described by Elo and Kyngäs (21). First, the audio-recorded interviews were transcribed in their original language. To obtain an overall picture of the interview, all transcribed interviews were read without any initial attempts to conduct coding by two independent researchers who had rich experience in qualitative data processing (HXM and YJM). The transcripts were then read and coded independently by two researchers to identify common patterns. These initial codes were further grouped into subcategories and categories. The subcategories and subsequent categories were discussed collectively with other members of the research team until a consensus was reached. Once no new categories emerged from the data, researchers re-examined the data and agreed upon a number of higher order categories. Selected quotes were translated into English to illustrate intrapersonal and interpersonal factors affecting the self-care practices among the individuals with COVID-19 in Hong Kong.

### Establishing rigor and trustworthiness

The rigor of the study was enhanced following Lincoln and Guba's four general criteria: credibility, transferability, dependability, and confirmability (22). To enhance credibility, participants were recruited from different age and gender groups. Also, credibility was achieved by immersion in the data and researcher triangulation. Three researchers (HXM, YJM, MC) read and analyzed the data first independently and then collectively. Two had a nursing background (HXM, MC) and one had experience in psychology and social work (YJM). Member checking was performed to seek additional feedback from the participants. Transferability was assured by a rich description of the context, details of study methods and characteristics of the participants. Dependability was assessed by developing an audit trial, which documented the steps in the data analysis procedure. Confirmability was achieved by peer debriefing within the entire research team.

### Ethical approval

Ethical approval was received from the Human Subjects Ethics Sub-Committee of Hong Kong Polytechnic University (HSEARS20220310002).

## Results

The characteristics of the 30 participants are shown in Table 1. They had a mean age of 55 (±12.8) years and were predominantly female (76.7%). One-third of the participants (33.3%) had received tertiary education, and two-thirds of them (66.7%) were married. Approximately half were unemployed or retired. Thirty percent of the participants had reported having a chronic disease/s. Most of the participants lived with their family members (83.3%) when testing positive for the virus. Three participants' family members moved to hotels or relatives' homes for fear of infection. Seventy percent of the participants had experienced family outbreaks of the COVID-19. Before they were diagnosed with COVID-19, 90% of them had received at least one dose of a recognized and approved COVID-19 vaccination.

**Table 1:** Characteristics of the participants.

**Characteristics**	***N* (%)/Mean (SD)**
**Age (years)**	55 (12.8) (range: 32–90)
Gender	
Male	7 (23.3%)
Female	23 (76.7%)
Education level	
Primary	5 (16.7%)
Secondary	15 (50.0%)
Tertiary	10 (33.3%)
Work status	
Employed	16 (53.3%)
Unemployed	7 (23.3%)
Retired	7 (23.3%)
Religion	
Buddhism	4 (13.3%)
Christianity	1 (3.3%)
Hinduism	2 (6.7%)
No religion	23 (76.7%)
Marital status	
Single	5 (16.7%)
Married	20 (66.7%)
Divorced	1 (3.3%)
Widowed	1 (3.3%)
Separated	3 (10.0%)
Vaccination status	
Yes	27 (90.0%)
One dose	4 (13.3%)
Two doses	18 (60.0%)
Three doses	5 (16.7%)
No	3 (10.0%)
Date of first positive test	
3^rd^ week of February	3 (10.0%)
4^th^ week of February	6 (20.0%)
1^st^ week of March	13 (43.3%)
2^nd^ week of March	5 (16.7%)
3^rd^ week of March	2 (6.7%)
4^th^ week of March	1 (3.3%)
Screening test for Covid-19	
Rapid antigen test (RAT)	23 (76.7%)
Polymerase Chain Reaction (PCR)	4 (13.3%)
Both	3 (10.0%)
Living condition	
Living alone	5 (16.7%)
Living with family	25 (83.3%)
No. of family outbreaks	21 (70.0%)
Total no. of cases among family outbreaks	57
Length of home quarantine (days)	10.8 (5.7)
Range: 3–28
History of chronic diseases	
Yes	9 (30.0%)
No	21 (70.0%)

The vast majority of the participants contracted COVID-19 during the months of February and March (80.0%). Most of them (76.7%) used the RAT to detect COVID-19. None of them required hospitalization. All participants managed their symptoms with paracetamol and/or traditional Chinese medicine (TCM) (e.g., Lianhuaqingwen) at home. Their average length of home quarantine was 10.8 (3–28) days.

Factors influencing participants' self-care practices during home quarantine were classified into either intra and/or interpersonal levels. The details of the main categories and subcategories are presented in the following section.

### Intrapersonal-level factor

The most frequently mentioned individual-level factors included socioeconomic status, housing conditions, information and knowledge about COVID-19, long COVID, and psychological adjustments brought about by home quarantine.

### Socioeconomic status and housing conditions

Socioeconomic status and housing conditions had a negative impact on self-care for individuals with COVID-19. From the perspectives of participants who were their families' breadwinners, making money was seen as more important than being infected with COVID-19. Some participants talked about people they knew who went back to work before being fully recovered or kept their condition secret from their employers. For example, one respondent commented that:

*A friend of mine is a construction worker and is paid daily. For fear of being required* [to] *quarantine, he kept his infection status from his boss and continued his work. (#3, female, 60y)*

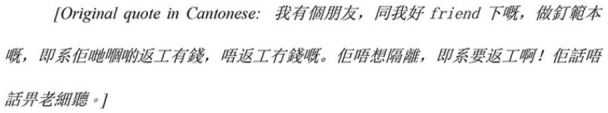

For many, the need and necessity to earn a living meant that they often exposed others to the virus. The threat of quarantine at a government facility for up to 3-weeks could result in a loss in income which caused concern for those family members who relied on the income to meet daily living costs. Equally, housing conditions were a major concern for most participants in implementing self-care at home. As most of them lived together with their family members in tiny flats (in most instances <400 square feet), they had experienced a high level of stress around the potential risks of spreading the virus to others. They rearranged their living conditions by staying in different rooms or a small isolation area which then added to their stress, especially when trying to follow strict hygiene and physical distancing practices. Despite great efforts, family outbreaks still occurred. Those who unintentionally spread the virus to others often experienced regret and guilt and blamed themselves for being the source of contagion.

The poorer participants who lived in cramped subdivided flats (a larger apartment that has been subdivided into 4–6 one-room apartments often with shared amenities such as kitchen and toilet) without elevators reported more challenges in dealing with self-care. The tiny space meant that they could hardly move around freely, let alone exercise. Being “trapped” in these tiny flats, participants had very little to do but lay in bed all day which increased feelings of being “irritable” and “helpless” with their current situation. In some cases, for those with limited mobility, simply navigating the stairs resulted in poor access to basic needs, such as food, resulting in a lower quality of life, for example:

*I live on the eighth floor of a subdivided flat without an elevator. There is no kitchen at home. After contracting the Covid-19, I felt too weak to go downstairs. However, most of the food deliverymen refused to climb the stairs. I survived on a few slices of bread those days. (#20, female, 58y)*

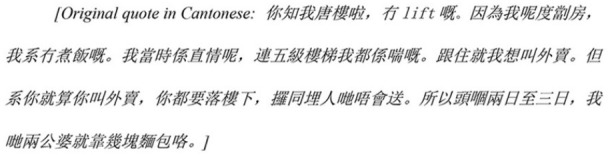

### Information and knowledge about COVID-19

Seeking information and knowledge related to implementing self-care for COVID-19 were commonly cited at the intrapersonal level. Many participants claimed that they had searched for information about the pandemic and self-care plans as well as stocking up sufficient daily necessities and medications in case of infection and home quarantine. The majority of the participants, especially those who experienced mild to moderate influenza-like symptoms, had confidence in managing the symptoms themselves by closely measuring their body temperature, having regular RAT tests, taking enough rest and fluid, and using over-the-counter (OTC) medications. Participants who had experienced severe symptoms also demonstrated sound knowledge and understanding in recognizing those symptoms that may require emergency care, for example:

*Although the cough was outrageous, I had confidence in managing my symptoms at home, because the experts said that the most important thing was monitoring the breath* [breathing] *and the oxygen saturation level and ensuring it was above 92%. I didn't experience shortness of breath, and I kept checking my oxygen saturation level with a pulse oximeter. It ranged from 93% to 97%. I knew I would be fine. (#29, female, 69y)*

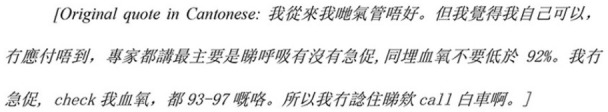

However, insufficient health care services information, such as supporting hotlines, self-reporting online systems, and health care services, were particularly challenging, leaving some participants feeling unsupported and stressed. For example, a participant complained about unclear information related to renal care during the peak of the outbreak:

*My mom has renal failure and usually has twice-weekly haemodialysis. It was so maddening when she contracted COVID. We had no idea where she could have the haemodialysis services. We just kept calling and calling all the numbers on the government website for help. (#20, female, 58y)*

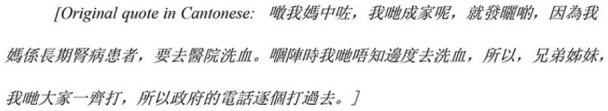

Furthermore, inadequate knowledge of self-medication was also reported. Many participants searched and applied various treatment regimens to improve their health, such as combining western medicine and TCM, together with supplements, and other remedies, which raised concerns about polypharmacy and medication errors, such as misuse, overuse, and potential side effects. For example:

*The experts and government officials recommended treatment with both western medicine and Lianhuaqingwen* [a TCM flu remedy]. *Thus, I followed their advice and asked my mom to take these medicines together. While I didn't know I should separate them by two hours. My mom got gastroesophageal acid reflux disease and was admitted to a hospital. (#25, female, 50y)*

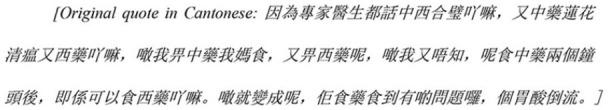

### Long COVID

Long COVID was an essential barrier to self-care. According to the National Institute for National Institute for Health and Care Excellence (NICE), the term “long COVID” refers to signs and symptoms that continue or develop after acute COVID-19 (symptoms for up to 4 weeks), includes both ongoing symptomatic COVID-19 (from 4 to 12 weeks) and post-COVID-19 syndrome (12 weeks or more) (23). Many participants, especially the older individuals, complained about impaired physical and cognitive functions as well as neuropsychiatric symptoms resulting from the long-term effects of COVID infection, which included shortness of breath, fatigue, brain fog, and insomnia. They felt their impaired psychophysiological functioning had severely impaired their abilities to perform daily tasks and were also linked to negative psychological outcomes. As one woman stated:

*I got the infection four weeks ago and now still have massive fatigue. I could hardly walk downstairs to buy food. It was so frightening when I realized that I could not walk as far as I did before. (#20, female, 58y)*

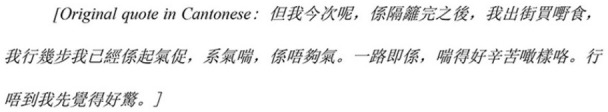

Another woman who was struggling with the symptoms associated with suspected brain fog stated:

*The brain fog symptoms are very irritating, and I cannot concentrate and forget things these days. It makes my everyday life overwhelming. Like I forgot I had switched off the fire after boiling a kettle of water when I was running into the kitchen. I don't know whether I could get recovered. (#16, female, 58y)*

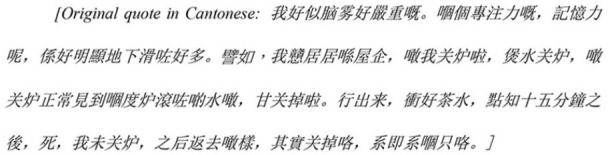

### Psychological adjustments brought about by home-quarantine

Home quarantine, inadequate information and resources, long COVID, and economic hardships induced a wide range of mental health issues among the participants. They consistently reported negative emotional responses to their situation, such as “scary,” “fearful,” “helpless,” “frustrated,” and “burnout.” For example:

*The virus messed me up. I felt so worried and depressed when I heard the news that COVID-19 may result in long-term symptoms and accelerated aging. I fear I will develop dementia soon, and I am scared of getting it again. (#10, female, 55y)*

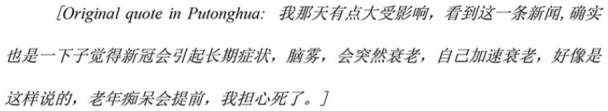

From some participants' perspectives, being infected with COVID-19 was a “shameful and dangerous” thing to disclose. To avoid social stigma, the participants, especially those suffering from long COVID (e.g., cough), preferred to hide their infection status and limit their contact with the outside world, even though they had tested negative for COVID-19. The fear of social stigma also induced a form of psychological stress and hindered their engagement in physical activities and other help-seeking behaviors:

*Although I test negative, my cough is still irritable and uncontrollable. I still stay at home and am afraid to go outside to exercise, because others will stigmatise me if I cough. (#29, female, 69y)*

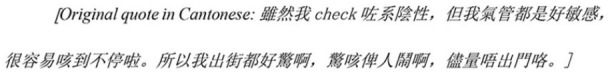

By contrast, some participants presented good psychological adjustment when confronted with social stigma and mental health issues. They employed various strategies to cope with their negative emotions, such as religious coping, acceptance, positive thinking, and attentional diversion.

*I believe in Buddhism. It keeps me mindful of my mental health. When I feel worried about the long COVID symptoms, mindfulness practice helps me calm down. (#10, female, 55y)*

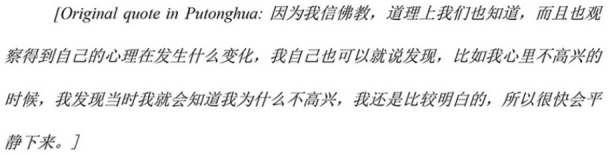

### Interpersonal level

Factors identified at the interpersonal level affecting self-care practices included caregiving responsibilities, family relationships, and social support.

### Caregiving responsibilities

Caregiving responsibilities was a key factor that influenced the participants in this study self-care practices at home, with many of them shouldering the responsibilities of taking care of children, parents, or significant others. Although they understood the importance of self-care and adequate rest after infection, they had to prioritize their family members' needs over their own. In many circumstances, they had to sacrifice their own health for the needs of their family. Feeling exhausted often affected their self-care practices and resulted in feeling stressed and depressed, an example of which was:

*I had many symptoms of COVID, but I needed to take care of my mom and my elder brother, cooking and cleaning. I felt extremely exhausted and burned out. (#17, female, 53y)*

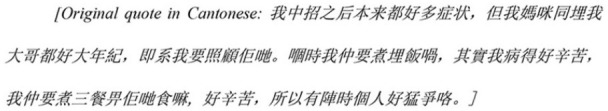

### Family relationships

Family relationships were portrayed as a key influencing factor for self-care. Participants with good family cohesion stated that the harmonious family relationship built upon their resilience in the face of adversity that COVID brought. To some extent, contracting COVID-19 strengthened their family bond and facilitated empathy and mutual support, one in which they experienced fewer mental health issues and felt more confident in managing their COVID-19 symptoms:

*We appreciate the infection, because it gave us an opportunity to spend time as a family and fight the challenges together. (#10, female, 55y)*.

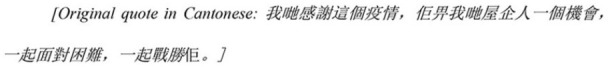

However, some participants experienced family conflicts after contracting COVID-19, because they “*blamed their family members for transmission of the virus*” or “*scolded their siblings for being unfilial*,” in other words, not fulfilling their obligations to care for their parents. These conflicts created more tension and stress for the participants. As an older participant described:

*I lived with my younger daughter, and both of us were infected with COVID-19. My younger daughter blamed her sister for being unfilial since she didn't come to take care of me. She also blamed me for loving her sister more. I felt so sad. (#28, female, 90y)*

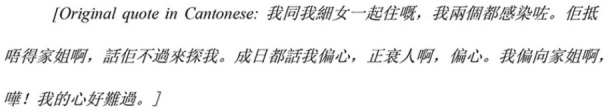

### Social support

Social support was a significant determinant of self-care at the interpersonal level, especially for these home quarantined individuals. The vast majority of the participants received information, COVID-19 specific material, emotional, and direct support from their social networks, such as family, friends, and peers:

*My parents dropped off groceries for us. The medicine, I mean, the clinic has a delivery service that I think that uses SF Express [a courier service] or something. But my dad also wanted to pick up the medicine for me and drop them off so we could use those courier delivery services. My parents did all the pickup and buying of food. Selfless. (Original quote in English) (#7, female, 40y)*

However, self-care concerns also arose among participants who lived alone, especially the older individuals. A lack of social support increased their sense of isolation and loneliness which reduced their access to help. As one participant described:

*An elderly friend of mine contracted the Covid-19, and I paid a visit to her. However, when I opened the door, I found she had fainted at home alone. It was so terrible. She cried when she woke up, “I called for help, but nobody came. I nearly died.” (#22, female, 40y)*

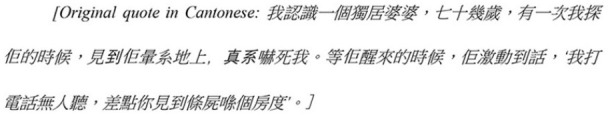

## Discussion

To the best of our knowledge, this is the first qualitative study that has explored self-care practices among Hong Kong individuals during the peak of the fifth wave of COVID-19. The participants' self-care experiences varied greatly, which were determined by various intra and interpersonal level factors. Among the reported factors, socioeconomic status, current housing conditions, information and knowledge of COVID-19, the effects of long COVID, caregiving responsibilities, and family relationships were considered imperative.

Findings from this study raise concerns about inequity in health and self-care practices among the socially and economically deprived populations, especially the poor and older adults. They experienced inequalities in accessing the essentials of daily living, medications, and appropriate and up-to-date health information. The suspension of social welfare services due to the pandemic further reduced their access to other services. Our findings align with results reported in previous studies on inequality and COVID-19 in that people of a low socio-economic status and older adults had been disproportionately affected by COVID-19 and were less likely to implement appropriate self-care activities (24, 25). Concerns for these marginalized and vulnerable groups need to be taken into account by governments departments and services when developing policies and supporting future epidemic/pandemic programmes. Although it might be considered a little late, it was good to see that the local government had made significant efforts to provide outreach services to the poor and the old people post-peak pandemic, by distributing anti-epidemic service bags and educating individuals on how to use the RAT kit. This was also strengthened by further efforts aimed at minimizing the risks of social exclusion/isolation, especially for those living alone and older adults.

Information and knowledge have been consistently described as intrapersonal-level factors that affect self-care practices across various populations (26, 27). This study also revealed that information and knowledge were essential factors in performing self-care practices among individuals with COVID-19 in Hong Kong. The increased information and knowledge of the virus individuals gained over the past 2 years, influenced their health risk perceptions and willingness to engage in risk reduction behavior, and also facilitated their self-efficacy and confidence in managing their symptoms (28, 29). However, a lack of information about health and supporting services was also reported. Insufficient knowledge of self-medication was particularly alarming, which was considered a predominant cause of polypharmacy and medication errors among these participants. This correlates with findings that a lack of clear information had led people to resort to self-medicating often with significant side effects (30). The participants' in this study self-medication practices could also be explained by various factors, such as lack of and conflicting treatment advice and evidence, limited access to health care services, and the negative emotions associated with the impaired function.

Long COVID was frequently mentioned among the participants and viewed as a significant barrier to maintaining adequate self-care. Currently, there is no consensus regarding the definition of long COVID. Findings from this study indicate that long COVID severely affected their day-to-day activities and triggered some mental health issues as well as the fear of social stigmatization that a positive COVID diagnosis would bring. A lack of evidence-based treatment options and the unpredictability of recovery further worsened their mental health status and contributed to increased feelings of frustration and helplessness. These findings are broadly consistent with those found in previous studies that COVID-19 survivors and those experiencing long COVID were more likely to have worsening mental health issues and quality of life (31–35). As the COVID-19 pandemic continues into its third year, long COVID has gained increasing recognition worldwide. Concern for the wide range of physical symptoms and mental health issues has resulted in a strong call for establishing long-COVID clinics with health care professionals from multidisciplinary backgrounds (36, 37). To better develop the health care service plan for people with long COVID in Hong Kong and other countries, large-scale epidemiological studies are recommended to better estimate the prevalence and outcomes of long COVID.

In this study, caregiving responsibilities placed a significant burden on the participants and contributed to their exacerbated poor health and inadequate self-care practices. This finding also is also corroborated by previous studies which suggested that the increased caregiving burden as a result of COVID-19 had significantly compromised caregivers' well-being and self-care practices (38, 39). During the COVID-19 pandemic, caregivers experienced additional stress as they struggled to cope with home quarantine measures, social isolation, restricted social connections, and suspended support services, along with increased dependence from their care recipients. Having contracted COVID-19 themselves further impeded their ability to provide effective care.

Studies of family relationships and self-care practice among other populations found that family cohesion was associated with adherence to treatment regimens, mental health, and quality of life (40, 41). In our study, family relationships were found to have a significant role in promoting self-care among the participants. A high level of family cohesion strengthened family resilience and empathy, while low family cohesion created family conflicts and mental health issues. Therefore, one of the key points highlighted from this study is that interventions to empower vulnerable families during tough times could alleviate some of the issues of non-cohesion in the family unit. For example, the family resilience theory suggests that family belief systems, organizational patterns, and communication are key processes in promoting family resilience, which could build and strengthen family bonds and help families cope with additional external stressors such as COVID-19 (42). Belief systems, which figured highly in this study, encompassed making meaning of adversity, maintaining a positive outlook, and transcendence and spirituality. Organizational patterns refer to flexibility, connectedness, and social and economic resources. Communication consists of clarity, open emotional expression, and collaborative problem-solving. Future research should consider the domains of key processes of family resilience when developing family resilience programs.

## Limitations of the study

This study has several limitations. First, the participants were dominated by middle to old-aged Chinese females, which may limit the transferability of the results to men, other age groups and those of other cultural backgrounds. Second, the study only focused on the experience of self-care among individuals testing positive for COVID-19 during the peak of the fifth and worst wave of the pandemic in Hong Kong. It is possible that individuals' self-care experiences could be different at other time points, for example when COVID-19 was first classified as a pandemic in late 2019. Third, being infected with COVID-19 was perceived as a stigmatized condition among some participants, and therefore the risk of social desirability bias may not be ruled out in their descriptions of self-care experiences based on this. While other forms of data collection such as telephone interviews, as used in this study, might minimize such risks.

## Implications for future research and education

To improve the medication literacy among individuals with COVID-19, clear treatment guidelines in relation to self-care practices for COVID-19 are paramount. Moreover, further studies are recommended to investigate self-medication practices, especially within a pandemic and along with the long-term health outcomes among people with COVID-19 who self-cared during those critical times. In addition, evaluating the effectiveness of integrating pharmacists and other health care providers in the process of self-medication may help improve medication literacy as well as reduce the psychological anxiety and stress among individuals with COVID-19.

Also, it is essential to promote self-care among the caregivers with COVID-19 with both emotional and practice support. Self-help or self-compassion interventions, such as mindfulness-based interventions, delivered via a digital platform, could be applied as a feasible and effective way of promoting the mental health of the caregivers (43). Furthermore, to assist the caregivers with Covid-19, practical support, such as food delivery services via a third party (e.g., NGOs), may be considered. Additionally, caregivers are recommended to prepare a contingency plan in case of unable to fulfill their responsibilities.

In addition, the role of family relationships provides insights into self-care and family resilience in the context of COVID-19. To support families overcome their life challenges, it is necessary to recognize the vital role of family relationships and foster stronger family resilience. Family-based resilience interventions aiming at strengthening the key processes of resilience, i.e., belief systems, organizational patterns, and communication, are strongly recommended.

## Conclusions

This study provides valuable insights into self-care practices among individuals with COVID-19 during the worse outbreak of the pandemic in Hong Kong. Intrapersonal and interpersonal level factors played significant roles in influencing their self-care practices. Findings from this study addressed the concerns from socially and economically deprived populations and caregivers as well as increased the awareness of insufficient knowledge of self-medication and COVID-19 symptoms management, especially among individuals with long COVID. As the pandemic resolves, future developments could include establishing interdisciplinary long COVID-19 clinics, which might provide comprehensive rehabilitation services. In addition, family-based resilience programs are recommended to empower vulnerable families and bolster their resilience during tough times.

## Data availability statement

The raw data supporting the conclusions of this article will be made available by the authors, without undue reservation.

## Ethics statement

The studies involving human participants were reviewed and approved by Human Subjects Ethics Sub-Committee of Hong Kong Polytechnic University. Written informed consent for participation was not required for this study in accordance with the national legislation and the institutional requirements.

## Author contributions

HM and MC conceived of and designed this study. HM was responsible for conducting the interviews and data transcription and drafting the article. HM, YM, and MC were involved in data analysis and data interpretation. YM, MC, SG, SW, and IZ provided critical comments and helped revise the manuscript. All authors have read and approved of the final version of the manuscript.

## Conflict of interest

The authors declare that the research was conducted in the absence of any commercial or financial relationships that could be construed as a potential conflict of interest.

## Publisher's note

All claims expressed in this article are solely those of the authors and do not necessarily represent those of their affiliated organizations, or those of the publisher, the editors and the reviewers. Any product that may be evaluated in this article, or claim that may be made by its manufacturer, is not guaranteed or endorsed by the publisher.
