# Supplementary material for: Intrapersonal and interpersonal level factors influencing self-care practices among Hong Kong individuals with COVID-19—A qualitative study
Source: Front Public Health. 2022 Aug 24;10:964944. doi: 10.3389/fpubh.2022.964944 (PMC9449417; doi:10.3389/fpubh.2022.964944)
Supplement: Supplementary file 1 [file Table_1.DOCX]

**Appendix I: Interview guide**

1. When did you test positive for COVID-19? (Probe: How did you get to know the diagnosis? What type of test did you perform? Any idea about the route of transmission?)
2. What symptoms did you have? (Probe: How did you manage these symptoms? What kind of medicine did you use? Where did you obtain the information related to symptoms management? How long did the symptoms last?)
3. Could you describe your self-care experience? (Probe: Which intrapersonal level factors challenged your self-care practice at home? Which intrapersonal level factors made the self-care practice easier? What kind of interpersonal level factors affected your self-care practice? And what kind of intrapersonal/family support you had helping you overcome the difficulties?)
4. Is there anything else you would like to share about your self-care experience during home quarantine?
